# Mining Activities in Iron Ore Areas Have Altered the Diversity and Functional Structure of Rhizosphere Bacterial Communities in Three Crops

**DOI:** 10.3390/microorganisms13122728

**Published:** 2025-11-29

**Authors:** Yanping Xu, Hao Ren, Ziping Zou, Guohua Shen, Yunfeng Zhang, Maoling Tan, Qiang Li

**Affiliations:** Key Laboratory of Coarse Cereal Processing, Ministry of Agriculture and Rural Affairs, Sichuan Engineering & Technology Research Center of Coarse Cereal Industrialization, School of Food and Biological Engineering, Chengdu University, Chengdu 610106, China

**Keywords:** high-throughput sequencing, rhizosphere soil, bacterial community diversity, mining area crops, iron ore mine

## Abstract

The mechanisms by which iron ore mining activities affect the surrounding rhizobacterial ecology remain unclear. This study employed 16S rRNA high-throughput sequencing to analyze the structure and function of rhizosphere bacterial communities associated with three local crops, *Musa basjoo* Siebold L., *Triticum aestivum* L., and *Amygdalus persica* L., in mining areas. It is noteworthy that in the iron mining area, the relative abundance of *Sphingomonas* and *Nitrososphaeraceae* in the soil has decreased. In contrast, the relative abundance of *Streptomyces* in the rhizosphere soil has increased due to mining activities. Pearson correlation analysis showed that the abundance of *Sphingomonas* is significantly positively correlated with the soil organic carbon content. Redundancy analysis (RDA) indicates that *Streptomyces* exhibits a significant positive correlation with soil titanium and pH content, while showing a negative correlation with iron and lead content.

## 1. Introduction

Microorganisms, as vital components of soil ecosystems, are widely distributed in rhizosphere soils and play an irreplaceable role in material cycling, nutrient transformation, and maintaining crop health [1,2,3]. Changes in the structure and function of rhizobacterial communities can directly reflect the health status of the soil environment and are often used as important indicators for assessing the degree of disturbance to soil ecosystems [4]. Under long-term acclimatization to the surrounding environment, rhizosphere bacterial communities develop unique physiological and metabolic mechanisms to resist external stresses. Some bacteria can reduce the toxicity of soil pollutants through biotransformation, adsorption, or immobilization, thereby providing potential microbial resources for soil remediation [5,6,7].

In recent decades, the global demand for metals has intensified, resulting in frequent mining activities. Research has found that the mining industry generates approximately 10 billion tons of tailings each year, accompanied by acid mine drainage (AMD), and this is continuously increasing [8]. Acid mine drainage contains heavy metals, that can cause serious environmental harm [9]. Mining activities in iron ore fields often lead to large amounts of heavy metals (such as manganese, cadmium, and zinc) [10]. This leads to the degradation of soil ecosystem functions, a decline in fertility, and disruption of rhizosphere microbial communities. Ecological restoration in mining areas is urgently needed, and the local dominant crops play an important role in restoring and reconstructing the microbial ecosystem. Studies have found that rhizosphere microorganisms can utilize the dominant local crops to promote the secretion of root exudates, attracting more stress-resistant and growth promoting microorganisms to assist in restoring the local rhizosphere microecology [11,12].

Wuding County in Chuxiong Yi Autonomous Prefecture, Yunnan Province, possesses exceptionally abundant mineral resources [13]. While the mining industry has driven local economic income, mining activities have also left behind a large number of abandoned mines. The area features a typical plateau mountain climate with a relatively fragile soil ecosystem. Three crops *Musa basjoo* Siebold L., *Triticum aestivum* L., and *Amygdalus persica* L. are important locally. Contaminants remaining from iron ore mining operations significantly disrupt the rhizosphere bacterial communities of these three crops, posing potential risks to agricultural product quality and human health [14,15].

Perennial herbaceous plants *Musa basjoo* Siebold L., monocotyledon plant *Triticum aestivum* L., and perennial woody plants *Amygdalus persica* L. are three crops that have been cultivated on a large scale locally for a long time, and account for more than 30% of the local crop planting area. These crops can grow in mined and polluted areas, and after mining, they develop stable physiological response mechanisms in the local soil environment. Their growth status can effectively reflect the true condition of soil nutrients and pollutant accumulation in the area. It is also worth mentioning that rhizosphere microorganisms play an important role in preventing toxic metals from transferring to the aerial parts of plants; otherwise, these metals may be ingested by animals and humans. However, the effects of mining on the recombination of rhizosphere bacterial communities in these crops and the response mechanisms of these bacterial communities remain unclear. The reasons for the tolerance of these three crops to polluted mining areas are also unknown. This study examines the following scientific questions: (1) How do elevated metal concentrations and reduced nutrient levels in mining-affected soils influence soil and rhizosphere microbial communities and their functions? (2) How do mining and non-mining areas differ in their effects on rhizosphere bacterial communities of three crop species? The aim was to reveal the patterns of mining impacts on rhizobacterial communities of different crops and to elucidate the response mechanisms of these communities to contaminated environments, providing valuable insights for ecological risk assessment and agricultural soil restoration in mining areas.

## 2. Materials and Methods

### 2.1. Study Site and Sample Collection

**Figure 1:**
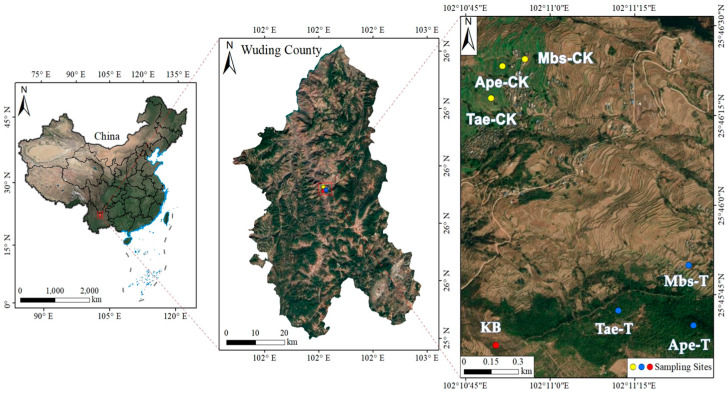
The study area is located in Wuding County, Chuxiong Yi Autonomous Prefecture, Yunnan Province. Mbs-T (*Musa basjoo* Siebold L.) rhizosphere soil in the mining area; Tae-T (*Triticum aestivum* L.) rhizosphere soil in the mining area; Ape-T (*Amygdalus persica* L.) rhizosphere soil in the mining area; Mbs-CK, rhizosphere soil in the non-mining area; Tae-CK, rhizosphere soil in the non-mining area; Ape-CK, rhizosphere soil in the non-mining area; KB, non-rhizosphere soil in the non-mining area.

The sampling site is located in Wuding County, Chuxiong Yi Autonomous Prefecture, Yunnan Province (101°55′ E to 102°29′ E, 25°20′ N to 26°11′ N), as shown in Figure 1. The area has an elevation ranging from 862 m to 2956 m and belongs to the subtropical low-latitude plateau monsoon climate zone, with an average annual temperature of 14.8 °C to 21.9 °C and an annual average rainfall of 800 mm to 1000 mm. The soil types are mainly red soil and purple soil, and the surrounding vegetation mainly consists of local crops and natural shrubs. The experimental group selected subsoil from the root zone of iron ore mining areas that had not been exposed, with the control area being natural topsoil unaffected by mining disturbance (cultivated layer thickness 15 to 20 cm). Before sampling, the sampling tools were disinfected with 75% alcohol and air-dried. To avoid direct contact with the samples, sterile gloves were worn to carefully dig out the entire root system of the crops, gently shake off the loose soil on the surface of the roots, and collect the soil closely adhering to the roots (usually < 2 mm) as rhizosphere soil.

Rhizosphere soil samples were collected from three plant species: Tae-T (*Triticum aestivum* L.), Mbs-T (*Musa basjoo* Siebold L.), and Ape-T (*Amygdalus persica* L.), both at the mine site and approximately 3 km outside the mining area. These samples were named Tae-T, Mbs-T, and Ape-T, respectively. Control samples included rhizosphere soils from the same three plant species grown in a non-mining area with similar environmental conditions (designated as Tae-CK, Mbs-CK, and Ape-CK). Non-root-zone soil from non-mining areas with similar environmental conditions was designated as the control (CK). Three biological replicates were established, with each replicate sample derived from a distinct individual plant. At the time of sampling, approximately 50 g of rhizosphere soil was collected from each plant, sealed in sterile bags, and transported to the laboratory in a cooler with ice packs. All samples were processed within 24 h of collection.

### 2.2. Determination of Soil Physicochemical Properties

Soil samples were analyzed for pH, organic carbon, total potassium, total nitrogen, total phosphorus, available potassium, alkali-hydrolyzable nitrogen, available phosphorus, and multiple elements. The experiment included three groups of soil samples, with three replicates per group. Soil pH was measured in a suspension of dried soil and deionized water using a pH meter [16]. Organic carbon was determined by measuring soil organic matter using the potassium dichromate volumetric method with external heating [17]. Total potassium was measured by atomic absorption spectrometry [18]. Total nitrogen was determined using the Kjeldahl method [19]. Total phosphorus was measured by the NaOH alkaline fusion-molybdenum antimony spectrophotometric method [20]. Available potassium was determined using NH_4_OAC extraction and flame photometry [21]. Alkali-hydrolyzable nitrogen was measured by the alkaline diffusion method [22]. Available phosphorus was determined using the molybdenum blue colorimetric method [23]. The determination of five metals, copper, zinc, iron, titanium, and lead, was carried out using inductively coupled plasma atomic emission spectroscopy.

### 2.3. PCR Amplification and Detection

Soil samples were transported refrigerated at 4 °C and arrived at the laboratory within 24 h for processing. Take 5 g of rhizosphere soil from each sample for DNA extraction and 16S rRNA gene sequencing. Genomic DNA was extracted from the soil samples using a specialized soil DNA extraction kit (D5625-02, manufactured by OMEGA, located in Los Angeles, CA, USA). After extraction, monitor the quality of the DNA on a 1% (*w*/*v*) agarose gel. The extracted and prepared DNA was diluted with sterile water to a concentration of 1 ng/µL Following dilution, the 16S rRNA gene V3–V4 region was amplified using primers incorporating barcodes (341F: 5′-CCTACGGGAGGCAGCAG-3′; 806R: 5′-GGACTACNVGGGTW-TCTAAT-3′) [24]. Following PCR completion, the products were thoroughly mixed with buffer and subjected to electrophoresis on a 2% (*w*/*v*) agarose gel. Upon amplification, the PCR products were purified using the Qiagen Gel Extraction Kit (Qiagen, Hilden, Germany). The sequence data has been deposited in the NCBI Sequence Read Archive under the accession number PRJNA1369752.

### 2.4. Library Preparation, Sequencing, and Data Processing

Libraries were constructed using the TruSeq^®^ DNA PCR-Free Sample Preparation Kit (Illumina, San Diego, CA, USA) according to the manufacturer’s instructions. Library quality was assessed using a Qubit fluorometer (Thermo Scientific, Waltham, MA, USA) and the Agilent Bioanalyzer 2100 system. Qualified libraries were then sequenced on the Illumina NovaSeq platform. Sequencing reads were demultiplexed based on their unique barcodes, followed by the removal of barcode and primer sequences. Paired-end reads were merged using FLASH V1.2.11 [25]. Raw tags were filtered according to the QIIME2 V202006 quality control pipeline to obtain high-quality tags [26]. The tags were aligned against the Silva reference database, and chimeric sequences were removed [27].

### 2.5. Noise Reduction and Species Annotation

The DADA2 V1.24.0 method within QIIME2 V202006 software was applied for noise reduction [28]. After denoising the valid tags, sequences with a count of less than 5 were filtered out, generating Amplicon Sequence Variants (ASVs) [29]. Based on the ASV annotations, the data was classified into species richness tables at the phylum, class, family, and genus levels. Relative abundance analysis of species was conducted, followed by clustering. Shared and unique ASVs among groups were identified using a Venn diagram [30].

### 2.6. Data Analysis

Alpha diversity was used to analyze the diversity of the microbial communities within samples, including seven diversity indices: observed_OTUs, Chao1, Goods_coverage, Shannon, Simpson, and Pielou_e [31]. Additionally, visualizations such as rarefaction curves and species accumulation boxplots were generated. Beta diversity was commonly used to analyze and compare the complexity of microbial communities among different samples. Beta diversity was calculated using QIIME2 V202006 to determine the Unifrac distances between samples [32]. Differences in beta diversity indices between groups were analyzed using Principal Coordinate Analysis (PCoA) and Non-metric Multidimensional Scaling (NMDS) to identify variations among different samples. Functional predictions were performed on the rhizobacterial communities of the three crops. PICRUSt2 was used to infer the potential functional capabilities of rhizobacteria [33], combined with the Gene Ontology (GO) [34], and the Kyoto Encyclopedia of Genes and Genomes (KEGG) databases [35].

### 2.7. Statistical Analysis

The significance of differences between samples was analyzed using statistical methods. For comparisons between two groups, a *t*-test was employed, while for comparisons involving more than two groups, Tukey’s test was used. A *p*-value of less than 0.05 was considered statistically significant.

## 3. Results

### 3.1. Determination and Physicochemical Analysis of Multiple Elements in the Rhizosphere Soil

**Figure 2:**
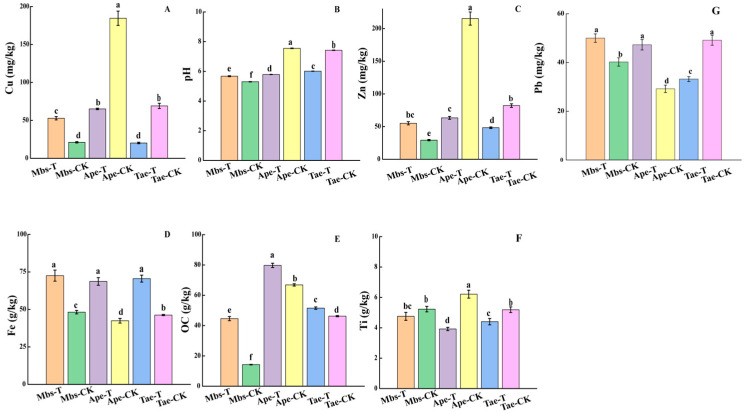
Metal element concentrations in rhizosphere soil samples ((**A**) (Cu); (**B**) (pH); (**C**) (Zn); (**D**) (Fe); (**E**) (OC); (**F**) (Ti); (**G**) (Pb)). Different letters indicate significant differences between samples (*p* < 0.05). Mbs-T (*Musa basjoo* Siebold L.) rhizosphere soil in the mining area; Tae-T (*Triticum aestivum* L.) rhizosphere soil in the mining area; Ape-T (*Amygdalus persica* L.) rhizosphere soil in the mining area; Mbs-CK, rhizosphere soil in the non-mining area; Tae-CK, rhizosphere soil in the non-mining area; Ape-CK, rhizosphere soil in the non-mining area.

As seen in Figure 2, compared with the controls, the Fe content in the rhizosphere soil of all experimental groups was significantly higher (*p* < 0.05), while the Ti content in the rhizosphere soil of the Ape-T and Tae-T experimental groups was significantly lower than that in their controls (*p* < 0.05); Cu and Zn were present in relatively low concentrations in the rhizosphere soil. Compared with the controls, the Cu and Zn contents in the rhizosphere soil of the Ape-T and Tae-T experimental groups were significantly lower (*p* < 0.05), while in the Mbs-T experimental group, the Cu and Zn contents were significantly higher than those in the Mbs-CK control group (*p* < 0.05). Compared with the controls, the Pb content in the rhizosphere soil of the Ape-T and Mbs-T experimental groups was significantly higher (*p* < 0.05). The Pb content in the Tae-T experimental group was significantly lower than that in the Tae-CK control group (*p* < 0.05).

Table 1 shows that the TK content in the rhizosphere soil of Mbs-T, Tae-T, and Ape-T plants was significantly higher than that in the control groups (Mbs-CK, Tae-CK, Ape-CK). However, the TN, AN, and TP contents in the rhizosphere soils of Ape-T and Tae-T were significantly lower than those in the control groups Ape-CK and Tae-CK (*p* < 0.05). Conversely, the AN, AK, TN, TP, and TK contents in the rhizosphere soil of Mbs-T were significantly higher than those in the Mbs-CK group.

### 3.2. High-Throughput Sequencing Data Analysis

**Figure 3:**
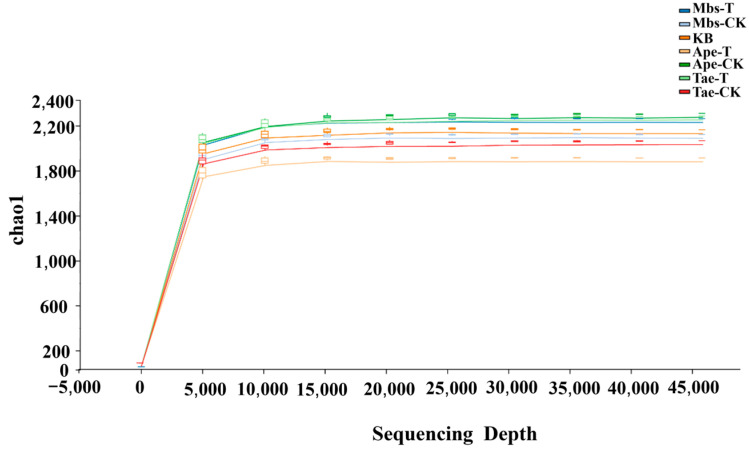
The dilution curves of the seven samples are shown. Mbs-T (*Musa basjoo* Siebold L.) rhizosphere soil in the mining area; Tae-T (*Triticum aestivum* L.) rhizosphere soil in the mining area; Ape-T (*Amygdalus persica* L.) rhizosphere soil in the mining area; Mbs-CK, rhizosphere soil in the non-mining area; Tae-CK, rhizosphere soil in the non-mining area; Ape-CK, rhizosphere soil in the non-mining area; KB, non-rhizosphere soil in the non-mining area.

Using 16S rRNA high-throughput sequencing technology, we investigated the bacteria associated with *Triticum aestivum* L., *Musa basjoo* Siebold L., and *Amygdalus persica* L. Batsch in both mining and nearby non-mining areas. As shown in Figure 3 (Table S1), the rarefaction curves of bacterial ASVs in different samples indicate that the number of observed species gradually increases with the increase in sequencing reads. The curves tend to flatten after the sequencing reads approach 15,000 indicating that the sequencing results are nearly saturated and the sequencing reads are sufficient to reflect the overall structure of the bacterial communities in the rhizosphere soil sample. The sequence abundance can be used to infer bacterial diversity in the samples.

### 3.3. Microbial Community Composition

Mbs-T (*Musa basjoo* Siebold L.) rhizosphere soil in the mining area; Tae-T (*Triticum aestivum* L.) rhizosphere soil in the mining area; Ape-T (*Amygdalus persica* L.) rhizosphere soil in the mining area; Mbs-CK, rhizosphere soil in the non-mining area; Tae-CK, rhizosphere soil in the non-mining area; Ape-CK, rhizosphere soil in the non-mining area; KB, non-rhizosphere soil in the non-mining area.

Across all samples, we identified 46 phyla, 111 classes, 407 families, and 819 genera. We compared the variation in the top 10 phyla and genera in terms of abundance.

**Figure 4:**
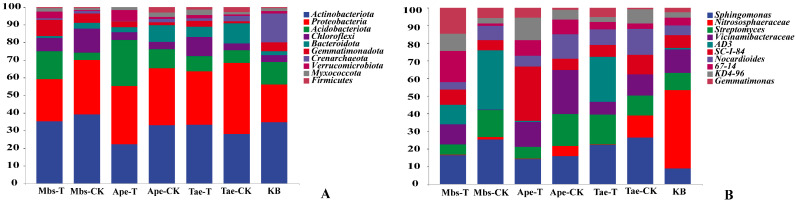
Relative abundance (top 10) of taxa at the phylum (**A**) and genus (**B**) levels in different samples. Each bar in the figure represents a sample size of 3 (*n* = 3), and the values shown are the mean relative abundances.

At the phylum level, we compared the top ten most abundant phyla across different samples, as shown in Figure 4A. *Actinobacteriota* are the most abundant at the phylum level, followed by *Proteobacteria*, *Acidobacteriota*, and *Chloroflexi*. Compared with the Mbs-CK samples, the Mbs-T samples exhibited decreased abundances of *Actinobacteriota*, *Chloroflexi*, and *Proteobacteria*, and a significantly increased abundance of *Acidobacteriota* (*p* < 0.05). Compared with the Ape-CK samples, the Ape-T samples showed a significantly decreased abundance of *Actinobacteriota* (*p* < 0.05), a decrease in *Proteobacteria* abundance, a significant increase in *Acidobacteriota* abundance, and an increase in *Chloroflexi* abundance. Compared with the Tae-CK samples, the Tae-T samples displayed increased abundances of *Actinobacteriota* and *Acidobacteriota*, a significantly decreased abundance of *Proteobacteria* (*p* < 0.05), and an increase in *Chloroflexi* abundance.

At the genus level, we compared the top ten most abundant genera across different samples, as shown in Figure 4B. *Sphingomonas* is the most abundant genus in horizontal samples, followed by *Nitrososphaeraceae, Streptomyces*, and *Vicinamibacteraceae.* Compared with the Mbs-CK samples, the Mbs-T samples exhibited a significant decrease in the abundance of *Sphingomonas* (*p* < 0.05), a decrease in the abundance of *Nitrososphaeraceae*, a significant decrease in the abundance of *Streptomyces* (*p* < 0.05), and an increase in the abundance of *Vicinamibacteraceae*. Compared with the Ape-CK samples, the Ape-T samples showed a decrease in the abundance of *Sphingomonas*, decreases in the abundance of *Nitrososphaeraceae* and *Streptomyces*, and a significant decrease in the abundance of *Vicinamibacteraceae* (*p* < 0.05). Compared with the Tae-CK samples, the Tae-T samples displayed an increase in the abundance of *Sphingomonas*, a significant increase in the abundance of *Streptomyces* (*p* < 0.05), a significant decrease in the abundance of *Nitrososphaeraceae* (*p* < 0.05), and a decrease in the abundance of *Vicinamibacteraceae*.

**Figure 5:**
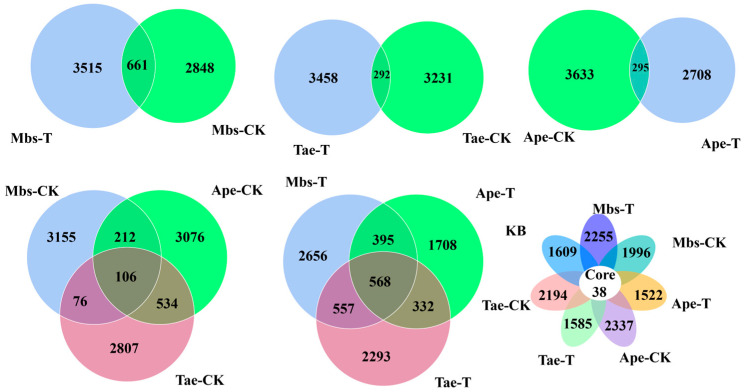
Venn diagrams and petal diagrams of the rhizosphere soils of different plants and nonrhizosphere soil samples from nonmining areas. Mbs-T (*Musa basjoo* Siebold L.) rhizosphere soil in the mining area; Tae-T (*Triticum aestivum* L.) rhizosphere soil in the mining area; Ape-T (*Amygdalus persica* L.) rhizosphere soil in the mining area; Mbs-CK, rhizosphere soil in the non-mining area; Tae-CK, rhizosphere soil in the non-mining area; Ape-CK, rhizosphere soil in the non-mining area; KB, non-rhizosphere soil in the non-mining area.

As shown in Figure 5 (Table S2), there were 661 shared ASVs between the Mbs-T and Mbs-CK samples, 292 shared ASVs between the Tae-T and Tae-CK samples, and 295 shared ASVs between the Ape-T and Ape-CK samples. Among the three plant species in the mining area, there were 568 shared ASVs, with 2656 ASVs unique to the Mbs-T samples, 1708 ASVs unique to the Ape-T samples, and 2293 ASVs unique to the Tae-T samples. Among the three plant species in the non-mining area, there were 106 shared ASVs, with 3155 ASVs unique to the Mbs-CK samples, 3076 ASVs unique to the Ape-CK samples, and 2807 ASVs unique to the Tae-CK samples. A total of 38 ASVs were common to all samples tested.

### 3.4. Analysis of Rhizobacterial Microbial Diversity

**Figure 6:**
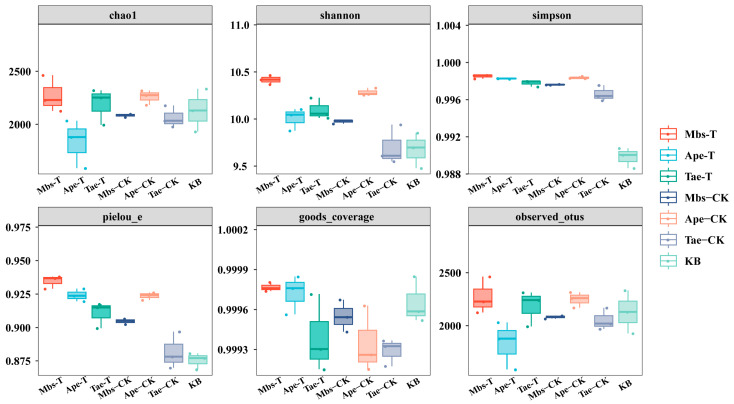
Box plot for the alpha diversity index of different samples. Mbs-T (*Musa basjoo* Siebold L.) rhizosphere soil in the mining area; Tae-T (*Triticum aestivum* L.) rhizosphere soil in the mining area; Ape-T (*Amygdalus persica* L.) rhizosphere soil in the mining area; Mbs-CK, rhizosphere soil in the non-mining area; Tae-CK, rhizosphere soil in the non-mining area; Ape-CK, rhizosphere soil in the non-mining area; KB, non-rhizosphere soil in the non-mining area.

The species diversity and distribution of rhizobacterial communities associated with the three plant species were analyzed using six alpha diversity indices, including observed_OTUs, Chao1, Goods_coverage, Shannon, Simpson, and Pielou_e, as shown in Figure 6. Different plants exerted varying influences on the bacterial diversity in their rhizosphere soil. Compared with plants grown in the non-mining area, the Mbs-T samples generally exhibited increased values for the observed_OTUs, Chao1, Goods_coverage, Shannon, and Pielou_e indices, while the Simpson index increased in both Mbs-T and Tae-T samples. Compared with plants grown in the non-mining area, the Ape-T samples showed a significant decrease in the Chao1 and observed_OTUs indices (*p* < 0.05), and a significant increase in the Goods_coverage index (*p* < 0.05). Compared with plants grown in the non-mining area, the Tae-T samples generally showed increased values for the observed_OTUs, Chao1, Shannon, Simpson, and Pielou_e indices, with the Shannon and Pielou_e indices showing a significant increase (*p* < 0.05).

**Figure 7:**
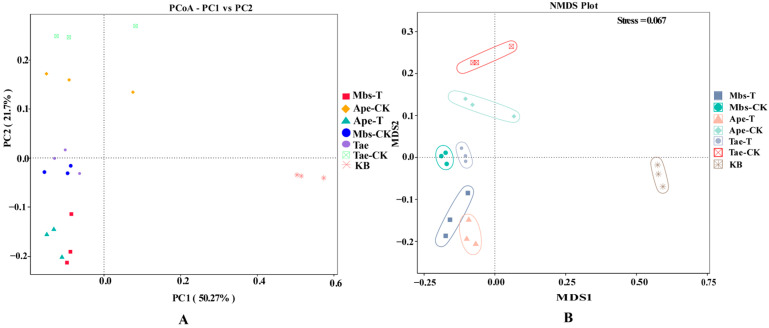
The weighted UniFrac distances of the different samples were determined by principal coordinate analysis (PCoA) (**A**) and nonmetric multidimensional scaling (NMDS) (**B**). Mbs-T (*Musa basjoo* Siebold L.) rhizosphere soil in the mining area; Tae-T (*Triticum aestivum* L.) rhizosphere soil in the mining area; Ape-T (*Amygdalus persica* L.) rhizosphere soil in the mining area; Mbs-CK, rhizosphere soil in the non-mining area; Tae-CK, rhizosphere soil in the non-mining area; Ape-CK, rhizosphere soil in the non-mining area; KB, non-rhizosphere soil in the non-mining area.

Principal Coordinate Analysis (PCoA) and Non-metric Multidimensional Scaling (NMDS) were conducted on our samples based on Weighted UniFrac distances. The impact of root-zone soil from mining areas on plant bacterial growth was assessed by evaluating differences formed among the three plant species. For instance, in Figure 7A (PCOA), significant differences emerged in the bacterial communities of the three plants compared to non-mining areas. Compared with non-mining area crops, the Mbs-T group showed smaller changes in bacterial communities across the three crop types. Significant differences were observed between the Ape-T and Tae-T samples. The non-metric multidimensional scaling analysis showed consistent results, further highlighting the differences between bacterial communities (Figure 7B).

**Figure 8:**
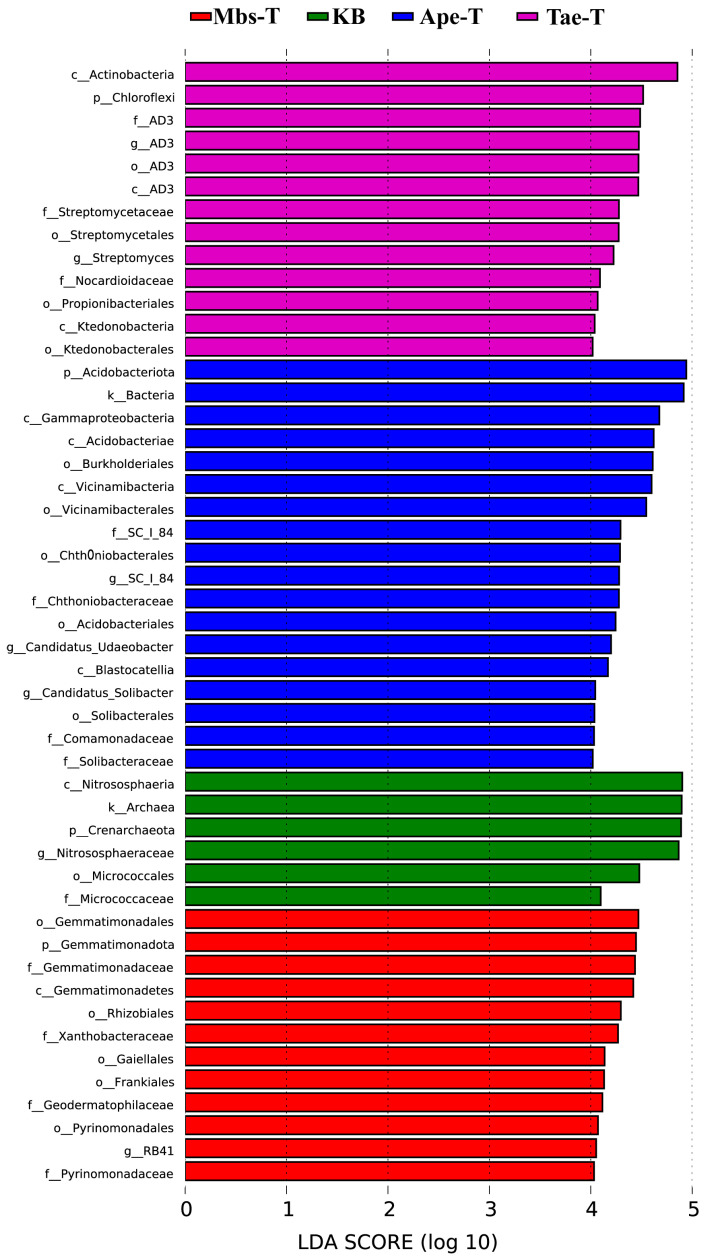
The LEfSe analysis compared the mining area group to the non-mining area group. Bacterial taxa significantly enriched from phylum to genus are identified with an LDA score > 4. The Mbs-T of the mining area is shown in red, Ape-T in blue, Tae-T in purple, and the non-mining area KB is shown in green. Mbs-T (*Musa basjoo* Siebold L.) rhizosphere soil in the mining area; Tae-T (*Triticum aestivum* L.) rhizosphere soil in the mining area; Ape-T (*Amygdalus persica* L.) rhizosphere soil in the mining area; KB, non-rhizosphere soil in the non-mining area.

As seen in Figure 8, the Tae-T rhizosphere bacterial community exhibits significant differences in the following groups: the phylum Chloroflexi, the class Actinobacteria, and the family AD3. On the other hand, the Ape-T rhizosphere bacterial communities encompass the phylum Acidobacteriota; the class Acidobacteriae also shows significant differences. The Mbs-T rhizosphere bacterial communities comprise the order Gemmatimonadales. In contrast, the non-mining area non-rhizosphere soil KB includes the phylum Crenarchaeota, and the class Nitrososphaeria.

### 3.5. Prediction of Rhizosphere Microbial Function

**Figure 9:**
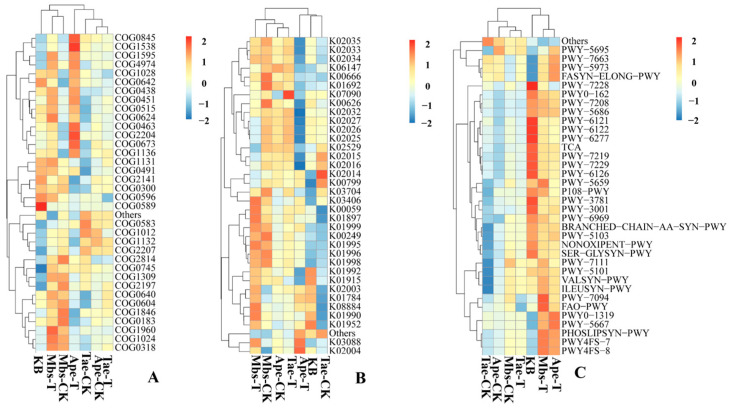
Prediction of KEGG (**A**), COG (based on the function of PICRUSt2 V2.5.2) (**B**), and MetaCycE (**C**). Different colors represent different relative abundances, with the increase in relative abundance represented by blue to red. Mbs-T (*Musa basjoo* Siebold L.) rhizosphere soil in the mining area; Tae-T (*Triticum aestivum* L.) rhizosphere soil in the mining area; Ape-T (*Amygdalus persica* L.) rhizosphere soil in the mining area; Mbs-CK, rhizosphere soil in the non-mining area; Tae-CK, rhizosphere soil in the non-mining area; Ape-CK, rhizosphere soil in the non-mining area; KB, non-rhizosphere soil in the non-mining area.

In the COG database shown in Figure 9A, compared with the Mbs-CK control group, the Mbs-T group exhibited enhanced function of acyl-CoA dehydrogenase associated with the alkylation response protein AidB (COG1960). Compared with the Ape-CK control group, the Ape-T group showed enhanced function of the DNA-binding transcriptional response regulator COG2204 and the outer membrane protein TolC COG1538. Compared with the Tae-CK control group, the Tae-T group showed decreased function of 2-succinyl-6-hydroxy-2,4-cyclohexadiene-1-carboxylate synthase MenH/undecaprenyl-phosphate glucose phosphotransferase UshA/YqjL COG0596.

In the KO database shown in Figure 9B, compared with the Mbs-CK control group, the functional expression of 3-oxoacyl-[carrier protein] reductase K00059 was enhanced in the Mbs-T group, while the functional expression of the predicted ABC transport system channel protein K02004 decreased. Compared with the Ape-CK control group, the functional expression of the predicted ABC transport system channel protein K02004 and RNA polymerase sigma-70 factor K03088 increased in the Ape-T group. Compared with the Tae-CK control group, the functional expression of uncharacterized protein K07090 was enhanced in the Tae-T group.

In Figure 9C, compared with the Mbs-CK control group, the Mbs-T group showed enhanced predicted functions in the PWY-7094 fatty acid salvage pathway and FAO-PWY fatty acid β-oxidation I. Compared with the Ape-CK control group, the Ape-T group showed enhanced predicted functions in PWY0-1319 CDP-diacylglycerol biosynthesis II and PWY-5667 CDP-diacylglycerol biosynthesis I. Compared with the Tae-CK control group, the Tae-T group showed reduced predicted function in FAO-PWY fatty acid β-oxidation I.

Figure 9 shows that three distinct rhizosphere communities may preferentially upregulate relevant functional genes to aid crop adaptation under environmental stress. The Mbs-T group COG1960, K00059, PWY-7094, and FAO-PWY, potentially linked to countering high Fe concentrations and potential organic pollution, thereby providing energy support for crop growth and repairing damage. The Ape-T group upregulates COG2204, COG1538, K02004, and PWY-5667, potentially reflecting adjustments made in response to information conveyed by the rhizosphere community to mitigate high Pb concentrations and low-nutrient stress. The Tae-T group exhibited downregulation of COG0596 and FAO-PWY, indicating suppressed basal energy metabolism and structural synthesis. This reflects heightened sensitivity to mining-related stresses and reduced rhizosphere bacterial diversity.

### 3.6. Correlation Analysis of Bacterial Communities in the Rhizosphere Soil of Three Plant Species

**Figure 10:**
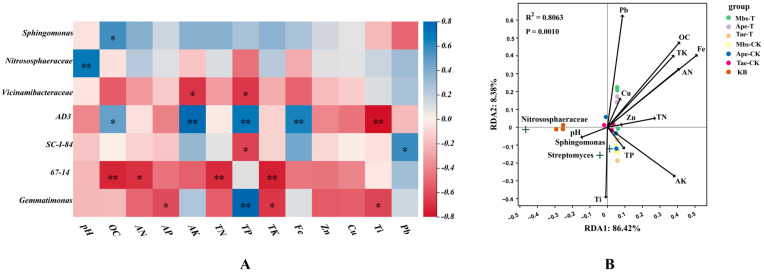
The physicochemical properties of the top 7 bacterial genera and soil were created as a heatmap based on the Pearson correlation algorithm. (**A**) The blue portion represents a positive correlation between soil physicochemical properties and bacterial abundance, whereas the red portion represents a negative correlation. An asterisk indicates that soil physicochemistry was significantly correlated with bacterial abundance (*p* < 0.01), and two asterisks indicate that the correlation was extremely significant (*p* < 0.05). (**B**) The redundancy analysis shows the relationship between the root-associated microbial communities and soil physicochemical factors. Black arrows represent different influencing factors, and the angles formed between different factors indicate their correlation: acute angles indicate a positive correlation, right angles indicate no correlation, and obtuse angles indicate a negative correlation. The longer the arrow, the greater the influence of that factor on the microbial communities.

From Figure 10A, the abundance of *Sphingomonas* is significantly positively correlated with OC content (*p* < 0.05). In addition, the abundance of *Nitrososphaeraceae* is significantly positively correlated with pH (*p* < 0.01). To explore the effects of key environmental factors on bacterial communities in rhizosphere soil, redundancy analysis (RDA) was performed, focusing on the relationship between the top ten genera and local soil environmental factors, as shown in Figure 10B. RDA1 and RDA2 explained 86.42% and 8.38% of the observed total variance, respectively, significantly elucidating the complex relationship between environmental factors. Compared to the control group, the Pb content in the rhizosphere of the experimental Mbs-T and Ape-T crops significantly increased (*p* < 0.05), while that of the Tae-T group significantly decreased (*p* < 0.05). This demonstrates the ability of Tae-T crop roots to translocate Pb factors and microbial community composition. Iron (Fe) was a major driving element. The *Nitrososphaeraceae* were significantly positively correlated with soil pH and significantly negatively correlated with Fe, Pb, and AN in the soil. *Sphingomonas* was positively correlated with soil TP, AK, and Zn content. *Streptomyces* was significantly positively correlated with soil Ti and pH.

## 4. Discussion

### 4.1. Impact of Mining Activities on Surrounding Soil

The physicochemical properties of soil are influenced by the surrounding environmental factors. A healthy ecological microbial community can also regulate soil health, while a contaminated environment can alter soil physicochemical properties [36]. Our study found that the soil pH associated with the crops was slightly alkaline or neutral. Compared with the control groups, the growth of the three crops in the experimental groups promoted an increase in the contents of OC, TK in the rhizosphere soil, likely due to enhanced interactions between crop root exudates and microorganisms [37], which accelerated the cycling and utilization of soil nutrients, thereby improving soil fertility. Furthermore, variations in heavy metal contamination were observed across different crops. Compared to the control group, the experimental group exhibited a significant increase in iron content across all three crops (*p* < 0.05). This indicates that bacterial activity within the rhizosphere soil of these crops may generate metabolic by-products such as organic acids and amino acids, which effectively solubilize iron within the soil. Moreover, iron serves as a key enzyme component for numerous microorganisms. In iron-rich environments, iron-reducing bacteria can facilitate the release of phosphorus from the rhizosphere soil for crop uptake [38]. Elevated Fe levels in the rhizosphere soil positively influence the local soil ecosystem stability and plant growth [39]. This aligns with our multi-element analysis of the rhizosphere soil. Research indicates that the root systems of certain crops possess the capacity to translocate Pb. Compared to the control group, the Pb content in the rhizosphere of the experimental Mbs-T and Ape-T crops significantly increased (*p* < 0.05), while that of the Tae-T group significantly decreased (*p* < 0.05). This demonstrates the ability of Tae-T crop roots to translocate Pb [40].

### 4.2. Effects of Mining Activities on the Composition of Rhizosphere Bacterial Communities

The mining activities in the Wuding County, Chuxiong Yi Autonomous Prefecture, Yunnan Province have led to alterations in the diversity and abundance of bacterial communities within the rhizosphere soils of Ape-T, Mbs-T, and Tae-T crops. These findings suggest that mining operations may have disrupted the original equilibrium of soil physicochemical properties, with metal contaminants resulting from extraction exerting a detrimental impact on the structure of rhizosphere bacterial communities. Furthermore, at the genus level, compared to non-mining area crop samples, the mining area exhibited higher abundances of *Sphingomonas*, *Nitrososphaeraceae*, *Streptomyces*, and *Vicinamibacteraceae* in the rhizosphere soil samples of the three crops. These bacterial groups can improve the environment, suppress pests and diseases, and regulate soil physicochemical conditions [41,42,43]. *Sphingomonas* exhibited a low abundance in rhizosphere soil samples from all three crops grown in mining areas. These rhizosphere bacteria secrete gibberellins and indoleacetic acid to enhance crop growth under stress conditions. Research indicates that *Sphingomonas* sp. LK11 plays a significant role in environmental remediation [44].

Communities of *Nitrososphaeraceae* can oxidize ammonium in soil, which is difficult for crops to utilize directly, into nitrate that plants can absorb. This aids crops in maintaining their nitrogen nutrition. The abundance of *Streptomyces* in the Tae-T samples increased significantly. Research indicates that *Streptomyces* exerts positive effects in contaminated soils, potentially through the roots of Tae-T crops synthesizing iron carriers or secreting growth hormones such as indoleacetic acid (IAA) and 1-aminocobaltopropane-1-carboxylic acid deaminase (ACCD) under *Streptomyces* induction [45,46,47]. Studies have also demonstrated *Streptomyces*’ high resistance to heavy metals such as mercury and nickel. Consequently, further development and utilization of this bacterium for mine soil remediation warrants consideration [48].

### 4.3. Effects of Mining Activities on the Diversity and Functional Differentiation of Crop Rhizosphere Bacterial Communities

For the three rhizosphere microbial strains Mbs-T, Tae-T, and Ape-T, we observed a promoting effect on the Chao1 index. This indicates that under heavy metal stress conditions, a positive microbial community succession is driven, enhancing symbiotic and interactive relationships among the bacterial communities in the plant rhizosphere [49]. Consequently, the differentiated rhizosphere bacterial communities better adapt to post-mining environments and maintain long-term community stability. Conversely, the elevated Pielou’s index in Mbs-T and Tae-T crops may relate to heavy metal impacts on rhizosphere bacterial communities, eliminating certain intolerant rhizosphere bacteria and promoting uniform distribution of tolerant taxa. This indicates that both crops possess adaptability to heavy metal stress. Notably, Mbs-T exhibited the highest Pielou’s index among the three crops. Further analysis of the Simpson diversity index suggests that the unique root exudate characteristics support heavy metal-tolerant microbial consortia. This may be involved in supporting heavy metal-tolerant microbial consortia. Mbs-T crop roots secrete organic acids that regulate soil pH, producing soluble sugars as carbon sources. These attract rhizosphere bacteria, such as *Sphingomonas*, that tolerate heavy metal stress and enhance crop growth [50,51]. Pearson correlation analysis revealed a positive association between *Sphingomonas* and organic carbon, further confirming that the abundance of rhizosphere bacteria is primarily influenced by the combined effects of soil physicochemical properties and heavy metals [52].

Rhizosphere soil bacteria, as central participants in the plant–soil system, play a crucial role in material cycling and stress responses. They exhibit functional differentiation within their communities that directly reflects environmental pressures reshaping the rhizosphere microbiome. We predicted the functional composition of rhizosphere bacterial communities in both mining and non-mining areas based on three databases: prediction of KEGG, COG, and MetaCycE. Significant differences emerged in multiple key functions between mined and unmined sites. Mining activities typically involve metal enrichment and nutrient imbalances; the upregulation of rhizosphere bacterial community functions may facilitate self-maintenance and stress adaptation.

Compared to the functional profiles of rhizosphere bacterial communities in non-mineralized zones, the functions of outer membrane protein TolC (COG1538), DNA-binding transcriptional response regulator (COG2204), putative ABC transport system permease protein (K02004), and RNA polymerase sigma-70 factor (K03088) all showed significant increases (*p* < 0.05). Notably, the outer membrane protein TolC’s function suggests that rhizosphere bacteria may produce iron carriers and indoleacetic acid to assist in concentrating heavy metal ions and toxic metabolites within rhizosphere bacterial communities before excreting them into the external environment, thereby sustaining survival [53]. DNA-binding transcriptional response regulators frequently interact with histidine kinases, acting as pivotal proteins regulating gene expression and controlling key gene activity. In metal-contaminated environments, the regulators may modulate critical gene expression, release detoxification signals, and maintain equilibrium within the mining area [54]. The putative ABC transport system permease protein exhibited a significant increase; in mediating metal transmembrane transport [55,56].

Under conditions of high metal concentrations (Fe, Pb) and low nutrient levels (TN, TP) in mining areas, root-associated bacteria face dual stressors. The rhizosphere microbial communities respond by remodeling their structure to regulate the soil environment and mitigate heavy metal stress. Originating from the selection and enrichment of soil bacteria within the plant rhizosphere, root exudates secrete sugars, metabolites, and nutrients for bacterial growth even under environmental stress, thereby recruiting more functional bacteria to exert their effects [57]. Compared to the control group, the iron content significantly increased in all three crops. This growth was linked to root exudate-mediated recruitment of iron-reducing bacteria. The LEfSe analysis revealed an enrichment of *Streptomyces* in the Ape-T rhizosphere crops, potentially mitigating metal toxicity by biosorbing Fe and Pb [58]. Redundancy analysis further confirmed negative correlations between *Streptomyces* and Pb, Fe, and TN. Compared to the control group, the Pb content in Tae-T was significantly reduced, potentially linked to the enrichment of *Sphingomonas* in Tae-T crops. *Sphingomonas* possesses multifunctional capabilities, enabling adaptation to diverse contaminated environments [59]. These include 1-aminocyclopropane-1-carboxylic acid deaminase (ACCD), iron carrier production for chelating heavy metal ions, phosphorus solubilization to yield phosphorus essential for crop growth, and the secretion by crop root systems of carbon sources and energy required by the bacterium [60,61]. Upon establishing stable root-associated microbial communities, plants better adapt to their surroundings by absorbing harmful elements. Native plants exhibit enhanced compatibility with indigenous microbial populations, nourishing beneficial microbial communities through root exudates to form stable biological consortia.

Functional predictions based on PICRUSt in this study require validation through metagenomic or transcriptomic approaches in future research. This provides an accurate basis for elucidating response mechanisms following metal contamination involvement and metabolic pathways for post-contamination biodegradation in future bioremediation studies.

## 5. Conclusions

This study investigated the effects of mining activities at the Tae-T, Mbs-T, and Ape-T sites on rhizosphere bacterial community diversity, community composition, and function. Iron ore mining altered the soil physicochemical properties, resulting in a weakly alkaline pH and significantly elevated iron content in the mined soils. Nutrient responses (TN, TP, AK) in the rhizosphere soils also exhibited species-specific variations among crops. Mining activities reduced the Tae-T rhizosphere bacterial community diversity and altered the bacterial community structure, resulting in a decrease in the abundance of *Sphingomonas* and *Nitrososphaeraceae*. Functional prediction analysis indicated that rhizosphere bacterial communities adapted to the environment by enhancing heavy metal transport and stress response-related functions (e.g., COG1538, K02004), while functions related to organic pollutant degradation and metabolism were suppressed. The stress-tolerant properties of *Streptomyces* and other dominant bacteria offer suitable microbial candidates for ecological restoration in mining areas. By employing dominant functional strains of *Sphingomonas* to enhance the purification efficiency of heavy metal pollutants, and concurrently implementing agricultural management adjustments such as balanced fertilization to modulate the stress resistance of rhizosphere bacterial communities, sustainable synergistic development between ecological restoration and agricultural production in mining regions can ultimately be achieved.

## Figures and Tables

**Table 1:** The content of TN, TP, TK, AN, AP, and AK in the rhizosphere soil.

Element	Mbs-T	Mbs-CK	Tae-T	Tae-CK	Ape-T	Ape-CK
AN (mg/kg)	394.19 ± 2.09 d	172.53 ± 1.94 f	353.64 ± 1.67 e	439.99 ± 3.47 c	553.13 ± 3.42 b	746.04 ± 2.13 a
AP (mg/kg)	14.37 ± 0.79 e	21.71 ± 1.32 c	70.74 ± 0.87 a	18.10 ± 0.99 d	28.48 ± 0.20 b	18.60 ± 0.66 d
AK (mg/kg)	387.60 ± 3.88 d	331.10 ± 3.04 e	588.52 ± 4.56 b	526.59 ± 4.44 c	336.60 ± 3.29 e	838.69 ± 8.34 a
TN (g/kg)	2.39 ± 0.01 e	1.13 ± 0.01 f	3.14 ± 0.01 d	3.52 ± 0.04 b	3.25 ± 0.02 c	5.67 ± 0.01 a
TP (g/kg)	0.91 ± 0.01 d	0.77 ± 0.04 e	1.13 ± 0.01 c	1.63 ± 0.03 b	0.94 ± 0.01 d	4.94 ± 0.03 a
TK (g/kg)	20.20 ± 0.15 c	5.94 ± 0.18 f	23.08 ± 0.21 a	14.77 ± 0.17 d	20.81 ± 0.18 b	9.79 ± 0.16 e

Mbs-T (*Musa basjoo* Siebold L.) rhizosphere soil in the mining area; Tae-T (*Triticum aestivum* L.) rhizosphere soil in the mining area; Ape-T (*Amygdalus persica* L.) rhizosphere soil in the mining area; Mbs-CK, rhizosphere soil in the non-mining area; Tae-CK, rhizosphere soil in the non-mining area; Ape-CK, rhizosphere soil in the non-mining area; Different letters indicate significant differences between samples (*p* < 0.05).

## Data Availability

The original contributions presented in this study are included in the article/Supplementary Material. Further inquiries can be directed to the corresponding authors.

## Supplementary Materials

The following supporting information can be downloaded at: https://www.mdpi.com/article/10.3390/microorganisms13122728/s1, Table S1: Dilution curve of the sample; Table S2: ASV data table with taxonomic information for mining and non-mining areas.
